# Editorial: Insights in Environmental, Aviation and Space Physiology: 2022

**DOI:** 10.3389/fphys.2023.1278192

**Published:** 2023-09-04

**Authors:** Richard Boyle, Hanns-Christian Gunga

**Affiliations:** ^1^ Retired, St. Augustine, FL, United States; ^2^ Charité—Universitätsmedizin Berlin, Corporate Member of Freie Universität Berlin and Humboldt-Universität zu Berlin, Institute of Physiology, Center for Space Medicine and Extreme Environments Berlin, Berlin, Germany

**Keywords:** adaptation, extreme conditions, decompression sickness, locomotor training and performance, pulmonary edema, gravity, space travel

In 1998 over 75% of the world’s population lived within 500 vertical meters above sea level (Cohen and Small, 1998; Peacock, 1998). This variation is generally inconsequential to physiological systems and inhabitants traveling within these elevations rapidly acclimate. Only about 0.004% of the world’s population lives above 5000 m (0.53 atm or 0.537 bar), where the fall in atmospheric pressure reduces the oxygen molecules present, now at nearly one-half (∼11.2%) that at sea level, and thus reduces the driving pressure for gas exchange in the lungs (Tremblay and Ainslie, 2021). Extending further in elevation physiological responses and adaptation, say to high altitude flight and space travel, can only be achieved using specialized life support systems. Another extreme occurs below sea level. Due to ambient pressure and CO_2_ buildup a human subject on average can breathe air through a tube at a depth of less than 1 m underwater. Air can still be used to a depth of about 60 m in recreational diving using specialized equipment to regulate the pressured air from a tank. Below that level non-recreational open-sea dives to a record depth 534 m (“Hydra 8” program) have been performed using exotic heliox (helium and O_2_) and hydrox (hydrogen and O_2_) mixtures or even deeper, e.g. ∼700 m, using an atmospheric diving suit. Going a step further one diver, Theo Mavrostomos, spent 2 hours under 71.1 atm of pressure in an onshore hyperbaric chamber in a simulated dive to 701 m using hydreliox (exotic breathing gas mixture of helium, oxygen and hydrogen). Challenges to human habitation are mounting even at our “sweet” zone of sea level. These are such snapshots of what physiologists face in the laboratory and in the field to better query the physiological adaptations of life on Earth and in its extremes including underwater and in space.

The present Research Topic, “Insights into Environmental, Aviation, and Space Physiology”, presents five original studies focusing on decompression sickness (Karimpour et al.), locomotion training (Borzyhk et al.) and performance (Miyatsu et al.), and space-related travel (Clément et al. and Mu et al.); two reviews on pulmonary edema (Tetzlaff et al.) and space-related travel (Barkaszi et al.); and a perspective on gravity’s effect on biology (Narayanan). This volume starts of a more comprehensive examination targeting environmental, aviation, and space physiology. Later volumes will be directed to understand how climate extremes, among them especially temperature, are radically changing and adversely affecting once habitable zones on Earth and the physiological adaptation of organisms inhabiting these zones. The latter case is extremely important. We are on a track losing our so-called “climatic-niche” (Xu et al., 2020) due to climate change and physiologists as well as clinicians have to understand how this kind of challenge might impact human health and performance. At that point, the physiological-medical research in extreme environments becomes an economical, social, political Research Topic. Although all the articles in this volume provide significant insights into the physiology of organisms under extreme conditions in diverse environments, we have selected two articles that highlight these diversities from the physiological effects acquired under the water’s surface to the balance and performance of space travelers beyond the boundaries of Earth’s atmosphere.

The Karimpour et al. paper deals specifically with the detection of post-dive venous gas emboli (VGE) with new hand-held ultrasound devices in comparison to standard echocardiography. Occupational and recreational divers as well as caisson workers perform their work or sport by breathing compressed breathing gas. The problem is that the breathing gas is provided throughout the dive according to ambient pressure. As oxygen is metabolized, inert gases, such as nitrogen in the case of compressed air, diffuse into the blood and saturate tissue “compartments” throughout the dive while ambient pressure is increased and maintained. During ascent, the pressure gradient reverses, and the inert gas accumulated in the various compartments is released back into the circulation and exhaled. The gas is exhaled as dissolved gas in the plasma but can also accumulate as small bubbles in supersaturated compartments, like poorly perfused ligaments, cartilage and fat, or in the blood. Such bubble formation can be detected by ultrasound techniques. However, Karimour et al. found that currently, neither device tested would be able to replace standard echocardiography for VGE assessment. Therefore, the well-documented high individual variability in VGE formation and the need to do more research in this perspective for the development of better safety diving guidelines, still asks for better technical solutions.

The maintenance of normal physiological function in the space environment is a formidable challenge to engineers and scientists. Advances in technology allow the crew to survive in a spacecraft off of Earth’s surface for relatively short time intervals, but an astronaut’s responses to long-term weightlessness and the performance of mission-critical tasks are unresolved and the subject of this study. Can experiences gained by former exposures to space assist and even predict how an astronaut will adapt to the planned exploratory missions particularly after landing on a remote surface? This far-reaching question was asked by Gilles Clément and his team at Johnson Space Center (Clément G, Moudy SC, Macaulay TR, Bishop MO and Wood SJ (2022). Mission-critical tasks for assessing risks from vestibular and sensorimotor adaptation during space exploration (Front. Physiol. 13:1029161. doi: 10.3389/fphys.2022.1029161). Using both first-time flyers and experienced crew (one or more stays of 6 months on the International Space Station, ISS), functional locomotion tasks were conducted to challenge vestibular function at various times before launch, immediately after landing, and then at days after return. Despite the anticipated degradation of the astronaut’s balance and locomotion skill immediately after landing that can last up to a week, no significant difference across the two groups of astronauts was found for any of the motor behavior measures collected in the study. The authors correctly discuss the difficulty of a direct comparison of behavioral data obtained in earlier orbital missions aboard spacecraft such as the Shuttle with those from ISS. Aboard the ISS the astronauts rigorously exercise 10 h or more per week. This was not possible in Shuttle-era missions and resistive exercise devices we know today will likely not be available in autonomous exploratory missions. Although vestibular function is targeted in these tests, other factors will also be at play and thus “self-administered integrative countermeasure approaches” must be identified and validated.
